# PennPET Explorer: Human Imaging on a Whole-Body Imager

**DOI:** 10.2967/jnumed.119.231845

**Published:** 2020-01

**Authors:** Austin R. Pantel, Varsha Viswanath, Margaret E. Daube-Witherspoon, Jacob G. Dubroff, Gerd Muehllehner, Michael J. Parma, Daniel A. Pryma, Erin K. Schubert, David A. Mankoff, Joel S. Karp

**Affiliations:** 1 Department of Radiology, University of Pennsylvania, Philadelphia, Pennsylvania; 2 Department of Biomedical Engineering, University of Pennsylvania, Philadelphia, Pennsylvania; and; 3 KAGE Medical, Wayne, Pennsylvania

**Keywords:** PET, whole-body imager, human imaging

## Abstract

The PennPET Explorer, a prototype whole-body imager currently operating with a 64-cm axial field of view, can image the major body organs simultaneously with higher sensitivity than that of commercial devices. We report here the initial human imaging studies on the PennPET Explorer, with each study designed to test specific capabilities of the device. **Methods:** Healthy subjects were imaged with FDG on the PennPET Explorer. Subsequently, clinical subjects with disease were imaged with ^18^F-FDG and ^68^Ga-DOTATATE, and research subjects were imaged with experimental radiotracers. **Results:** We demonstrated the ability to scan for a shorter duration or, alternatively, with less activity, without a compromise in image quality. Delayed images, up to 10 half-lives with ^18^F-FDG, revealed biologic insight and supported the ability to track biologic processes over time. In a clinical subject, the PennPET Explorer better delineated the extent of ^18^F-FDG–avid disease. In a second clinical study with ^68^Ga-DOTATATE, we demonstrated comparable diagnostic image quality between the PennPET scan and the clinical scan, but with one fifth the activity. Dynamic imaging studies captured relatively noise-free input functions for kinetic modeling approaches. Additional studies with experimental research radiotracers illustrated the benefits from the combination of large axial coverage and high sensitivity. **Conclusion:** These studies provided a proof of concept for many proposed applications for a PET scanner with a long axial field of view.

Molecular imaging with PET offers the unique ability to noninvasively interrogate biologic processes through the detection of emitted photons from an administered radiotracer. Although technologic advances in the development of modern PET scanners have enabled the acquisition of diagnostic-quality images in under 10 min, these instruments remain inherently inefficient. Limited by a standard axial field of view (FOV) of less than 26 cm, commercial PET scanners detect about 1% of emitted photons and need to move through several bed positions to capture relevant anatomy (1–3). To overcome these limitations, we have come together as the EXPLORER consortium to develop whole-body PET imaging devices (4,5). As part of this effort, we have developed the PennPET Explorer, a whole-body PET imager (6).

Whole-body PET imagers provide unique advantages over commercial state-of-the-art PET scanners. With an extended axial FOV, sensitivity increases and detection of isotopically emitted photons from a larger detection area is more likely. The increased sensitivity could be leveraged for shorter scans or, alternatively, a decreased administered activity without a compromise in image quality. Although the tradeoff between administered activity and image quality is well established, the dramatic increase in sensitivity afforded by a whole-body PET imager opens the door to previously unthinkable possibilities such as PET images with essentially negligible radiation exposure or dynamic images of the whole body with high temporal resolution. Imaging isotopes such as ^68^Ga, whose activity is often limited by generator production, or delayed imaging with longer-lived isotopes such as ^18^F to study late kinetics (7), becomes feasible. Even more delayed imaging can be obtained for longer-lived radiotracers, such as ^89^Zr, to study slower biologic processes, including dosimetry and cell-tracking applications, despite its low positron yield. Whole-body coverage enables kinetic analysis of lesions outside a standard axial FOV and ensures the inclusion of large vascular structures for input functions. Finally, the potential for rapid imaging with low administered activities could enable consideration of PET for use in a broad spectrum of diseases not currently interrogated by PET. These expanded capabilities have both research and direct clinical applications (4,5).

To develop whole-body PET imagers and realize the benefits of such a device, the EXPLORER Consortium was formed in 2015. Two large-axial-FOV PET scanners have been borne out of this program: a 194-cm scanner developed by a team at the University of California, Davis, in collaboration with United Imaging Healthcare and a scanner developed at the University of Pennsylvania in collaboration with KAGE Medical and Philips Healthcare. The first human studies of the former system have been previously published (8). High-quality images were seen in a series of 4 healthy volunteers; the ability to image with a lower administered activity and at later time points was also explored. Herein, we discuss the first human studies of the second system, the PennPET Explorer, a prototype whole-body imager in a 3-ring configuration operating with a 64-cm axial FOV, which will soon be expanded to 140 cm.

In these initial human studies of the PennPET Explorer, hereafter referred to as PennPET, we sought to progressively test the capabilities of this whole-body imager. We first imaged healthy subjects, then clinical subjects with disease, and finally research subjects. Imaging protocols were tailored to study the performance of the PennPET in the context of specific clinical and research questions matched to the subject and radiotracer. This study was designed to demonstrate how the sensitivity of the whole-body imager can be leveraged to benefit specific applications depending on the particular imaging need. The prototype configuration has sufficient axial coverage to demonstrate proof of the concept that a long axial FOV has benefits, although expansion of the system beyond its current axial length will permit simultaneous imaging of all major organs with adequate sensitivity at the extremities.

## MATERIALS AND METHODS

### Scanner Characteristics

The general design of the PennPET whole-body imager has been previously described (9,10). Our companion paper provides additional details and describes initial testing of the system, performance measurements, and optimization for human imaging (6). Here, we briefly summarize the salient characteristics. The prototype configuration has 3 rings and an axial FOV of 64 cm. The instrument is based on a digital silicon photomultiplier developed by Philips Digital Photon Counting (11) with 1:1 crystal coupling, high count-rate capability (noise-equivalent count rate > 1,000 kcps at 40 kBq/cm^3^), and a 250-ps timing resolution. With 3 rings, we achieve a sensitivity of 55 kcps/MBq, about 9 times greater than that of a single ring. Other salient performance measures include a spatial resolution of 4.0 mm and energy resolution of 12%.

### Image Reconstruction

All data are acquired in singles list-mode format and sorted into a list of coincidence events; randoms are estimated from the delayed events, scatter is estimated using time-of-flight single-scatter simulation (12), and the data are reconstructed using time-of-flight list-mode ordered-subsets expectation maximization (13) (25 subsets) into 2-mm isotropic voxels for the body and a 576-mm transverse FOV. The list-mode algorithm includes optimized basis functions to suppress image noise while preserving signal; hence, no postfiltering is used.

To facilitate a direct comparison with the PennPET data for the first subject, the standard-of-care (SOC) data from the clinical PET/CT system were reprocessed with the same reconstruction tools as used for the corresponding PennPET data. However, for the 3 clinical scans presented, the SOC data were reconstructed with a smoother basis function and into 4-mm^3^ voxels as used in the clinic, rather than the 2-mm^3^ voxels used for the PennPET reconstruction.

For the presented proof-of-concept studies, the CT scan from the comparator commercial PET/CT device was used for attenuation correction but was not required for anatomic localization. Clinical subjects were scanned with their arms up; all other subjects were scanned with their arms down. Commercial software (MIM Software, Inc.) was used to perform rigid-body registration between the non–attenuation-corrected PET images from the PennPET device and the CT image, which was then transformed and projected to form the attenuation correction factors. To aid in the alignment, a flat pallet with indexing marks was used for most scans to facilitate reproducible subject positioning and permit the use of rigid-body registration. The flat pallet was not used for the 3 clinical scans, although the registration was satisfactory for data correction. When the PennPET device is expanded to 140 cm, an integrated CT scanner will be installed.

### Human Studies

These studies were approved by the University of Pennsylvania Institutional Review Board and performed under IRB 809476. All study participants gave written informed consent. Subjects were required to be at least 18 y old, and pregnant women were excluded. Three groups of subjects were recruited: healthy volunteers (“volunteer subjects”), patients who had clinical PET scans as part of their SOC medical treatment (“clinical subjects”), and subjects participating in other PET research studies with permission of the research study (“research subjects”). The overall protocol for each group is described below; details of specific subject studies are provided in the Results section and in Table 1.

**TABLE 1:** Subject and Study Details

								PennPET scan		Clinical scan	
Subject no.	Age	Sex	Subject type	BMI (kg/m^2^)	Height (cm)	Tracer	Injected activity (MBq)	Uptake time	Duration (min)	Uptake time	Duration (min)
1	62	F	Volunteer	26.5	164	^18^F-FDG	577	**1 h 27 min**	**20**	45 min	20
								**3 h 10 min**	**20**		
								**5 h 14 min**	**20**		
								**7 h 17 min**	**30**		
								**9 h 5 min**	**30**		
								**18 h 50 min**	**60**		
2	56	F	Volunteer	21.6	154	^18^F-FDG	559	**1 h 33 min**	**10**	57 min	15
								5 h 0 min	15		
3	79	M	Volunteer	22.9	170	^18^F-FDG	551	**10–40 min (dyn)**		1 h 9 min	15
								**1 h 44 min**	**20**		
								**4 h 21 min**	**30**		
								**18 h 40 min**	**60**		
4	79	M	Volunteer	23.3	170	^18^F-FDG	518	0–60 min (dyn)		NA	
								2 h 23 min	20		
								4 h 52 min	25		
5	60	M	Clinical	20.1	173	^18^F-FDG	496	**2 h 46 min**	**10**	60 min	15
								**4 h 12 min**	**10**		
6	28	M	Research	23.7	163	^18^F-NOS	218	**1 h 39 min**	**30**	0–60 min (dyn)	
7	29	F	Volunteer	19.3	177	^18^F-FDG	500	**0–60 min (dyn)**		3 h 0 min	15
								2 h 13 min	20		
								4 h 57 min	30		
								23 h 2 min	60		
8	58	F	Clinical	25.5	162	^68^Ga-DOTATATE	152	2 h 21 min	20	1 h 5 min*	10
								**3 h 32 min**	**20**		
9	48	M	Research	26.3	178	^18^F-FTP	226	**2 h 31–59 min (dyn)**		0–120 min (dyn)	
10	60	M	Clinical	20.3	175	^18^F-FDG	555	**1 h 46 min**	**20**	1 h 2 min	15
								4 h 7 min	30		

* Siemens Biograph mCT was used as clinical scanner. All other clinical scans were performed on Philips Ingenuity TF PET/CT.

BMI = body mass index; dyn = dynamic; NA = not applicable; NOS = nitrous oxide synthase; FTP = fluortriopride.

PennPET scans included in article are in boldface font. Total scan duration for these studies is listed, although some images represent shorter scans by subsampling of data.

All volunteer subjects underwent a comparator scan on a commercial PET/CT scanner (Ingenuity TF; Philips Healthcare). For ^18^F-FDG, the comparator scan was acquired with SOC clinical parameters (1.5–2 min/bed position depending on body mass index) about 60 (±15) min after intravenous administration of approximately 555 MBq (15 mCi) of ^18^F-FDG. The subjects were then escorted to the PennPET, where scans were acquired at a single bed position without reinjection of the radiotracer. With 64-cm axial coverage, the subjects were imaged from the vertex of the head through the abdomen. To simulate scans of shorter duration, list-mode data were subsampled. Delayed images, up to 10 half-lives after injection, were obtained for select subjects to study late kinetics and the ability to image at low activity.

To study the potential for dynamic whole-body imaging, 2 volunteer subjects received bolus injections of ^18^F-FDG during imaging on the PennPET. After an hour of dynamic imaging, delayed scans were obtained. For these subjects, the SOC scan was acquired after the dynamic scan on the PennPET. These studies illustrate the wide dynamic range in count-rate capability of the instrument, which includes capturing the time–activity curves for the blood input functions.

After the feasibility of human imaging with the PennPET had been established, the clinical subjects were imaged upon completing their SOC PET/CT scan. One study used ^18^F-FDG with the Ingenuity TF (Philips Medical), whereas another study used ^68^Ga-DOTATATE with the Biograph mCT (Siemens Healthineers). The research subjects were also enrolled into this companion study to acquire additional images on the PennPET after completion of their primary research imaging. One study imaged ^18^F-NOS (14), an imaging agent that targets the inducible form of nitric oxide synthase specific to inflammation; the subject was imaged on the PennPET from the vertex to the lower abdomen 2 h after injection. Another study imaged ^18^F-fluortriopride, an imaging agent for the dopamine D_3_ receptor; PennPET imaging centered on the upper abdomen after consumption of a fatty meal to stimulate gallbladder emptying, as dosimetry studies have demonstrated that the gallbladder wall receives the highest dose (15).

## RESULTS

We initiated human imaging in August 2018, and during this initial period of evaluation we have imaged 10 subjects with 4 different tracers. Subjects ranged from 154 to 178 cm in height, with a body mass index of 19.3–26.5 kg/m^2^. The demographics and scan details for all subjects are available in Table 1. Images were selected to highlight specific features of this whole-body imager. We describe results for each specific type of study below.

### Volunteer Subjects

**FIGURE 1.**
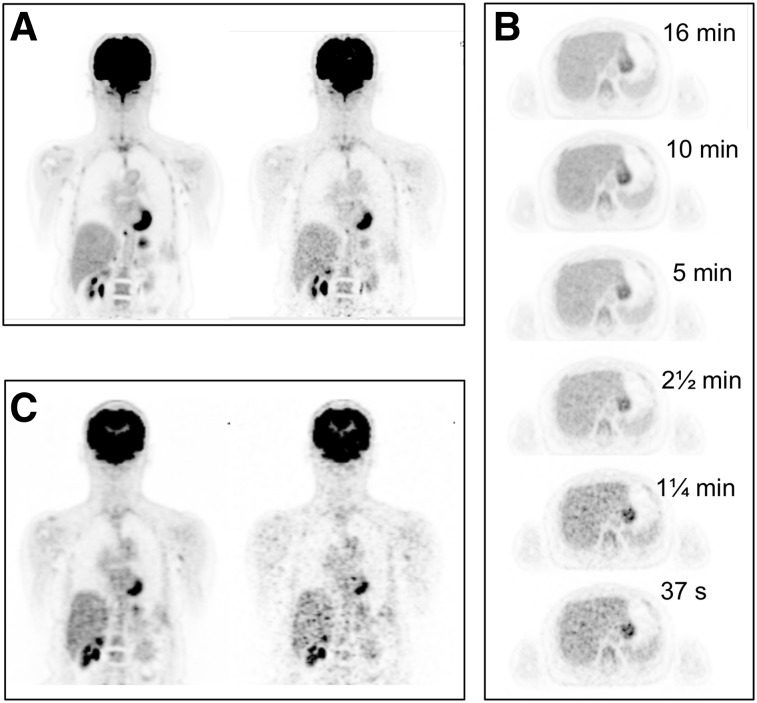
(A) ^18^F-FDG PET coronal images of subject 1 on PennPET acquired at 1.5 h after injection of ^18^F-FDG for 16 min (left) and 2 min (right). (B) Transverse images of liver from PennPET over range of scan durations. (C) Coronal images from SOC clinical PET acquired at 0.75 h after injection for 16 min (left) and 2 min (right).

Subject 1 was imaged several times on the PennPET, beginning 1.5 h after injection of ^18^F-FDG. The first PennPET scan was 20 min long. Data from both the PennPET and the clinical scan were subsampled and reconstructed to emulate shorter scans or, equivalently, lower activity. Reconstruction parameters were matched on both scanners to allow direct comparison. Image quality for this and subsequent studies was assessed by the 4 coauthors who are experienced clinicians with expertise in reading PET results. An image from a 16-min scan, chosen to match the clinical scan duration (for similar axial coverage), is shown in Figure 1A along with a subsampled 2-min scan. An image from the clinical scan is shown in Figure 1C along with a scan subsampled to 2 min. Qualitatively superior image quality—a combination of less noise and better anatomic detail—is seen in the 16-min PennPET scan compared with the clinical scan; the subsampled 2-min PennPET image demonstrates image quality comparable to, if not better then, that of the 16-min SOC clinical scan. In comparison, marked image degradation is seen in the subsampled 2-min clinical scan. The transverse slices of the PennPET data through the liver (Fig. 1B) illustrate the low noise and uniformity in the scans from 16 min to as short as 37 s (⅟_32_ subsampled data).

**FIGURE 2.**
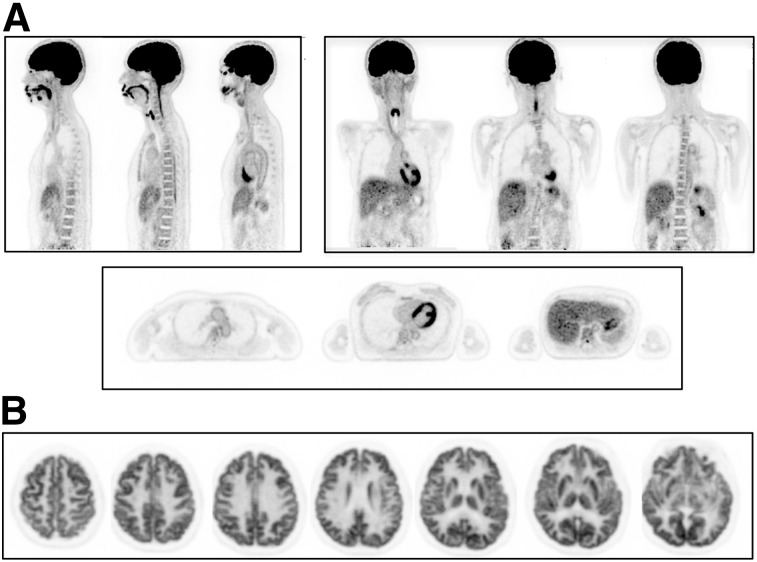
(A) ^18^F-FDG PET images of subject 2 (sagittal, coronal, and axial) on PennPET (10-min scan). (B) Transverse images on PennPET after subject was moved so that brain was positioned in center of axial FOV (10-min scan).

A 10-min ^18^F-FDG PET scan of subject 2 demonstrates the excellent image quality of the PennPET (Fig. 2A), as evidenced by the combination of low noise and structural detail (e.g., the vertebral bodies and vessel walls), as well as the ability to simultaneously image the brain and body. To fully demonstrate the structural detail of the PennPET, the subject was positioned with the brain centered in the axial FOV for a second scan. The transverse images through the cerebral hemispheres centered on the basal ganglia (Fig. 2B) demonstrate the high definition of these anatomic structures, as well as the high sensitivity of the instrument. We previously showed that brain images acquired near the center of the axial FOV have no evidence of spatial resolution blur, compared with images acquired near the edge of the axial FOV, despite the much larger acceptance of oblique lines of response (6). Combined with the high counts from being centered in the axial FOV, the PennPET could be used to better quantify radiotracer uptake and kinetics in these small structures, which have proven roles in neurologic disease.

**FIGURE 3.**
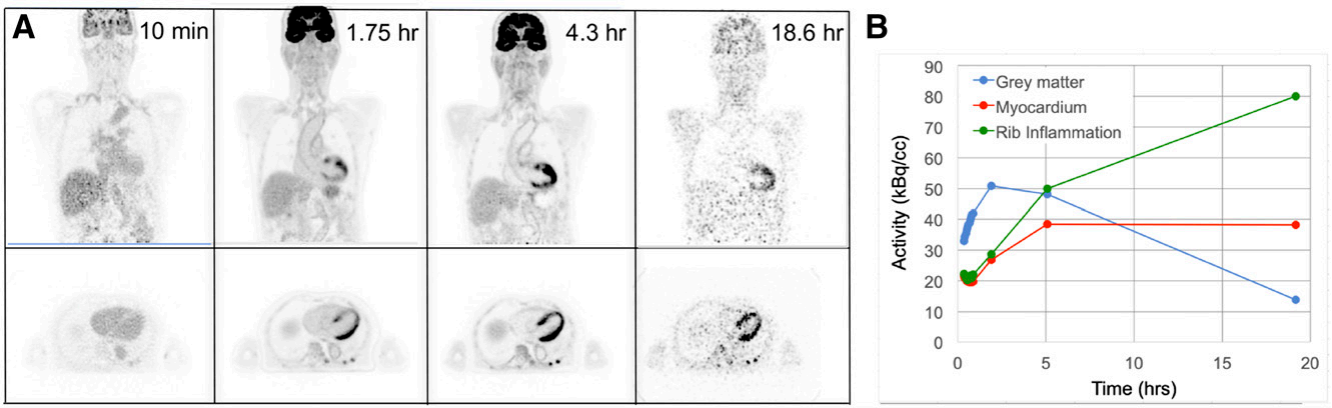
(A) ^18^F-FDG PET coronal images of subject 3 acquired at 4 time points after injection. First time point is 3-min scan; other time points are as noted in Table 1. (B) Time–activity curves for brain, myocardium, and rib fracture from same subject. Plotted points are at mid time of each scan.

Subject 3 was scanned dynamically on the PennPET at 10–40 min after injection of ^18^F-FDG; additional imaging was obtained out to 18.6 h after injection. The images in Figure 3A and the time–activity curves in Figure 3B illustrate the kinetics of normal ^18^F-FDG uptake over the entire imaging interval, demonstrating the potential to measure tracer kinetics over more than 10 half-lives of ^18^F. Blood-pool activity decreases over time, whereas ^18^F-FDG uptake in the myocardium increases. The 18.6-h delayed scan reveals decreased ^18^F-FDG uptake in the brain compared with earlier time points. Washout of ^18^F-FDG at such delayed time points has not been previously observed so clearly in humans. A subacute rib fracture demonstrates the expected increased uptake of ^18^F-FDG, which also increases over time relative to uptake in normal tissue. Similar kinetics for ^18^F-FDG on delayed images out to 10 half-lives (19 h after injection) were also measured for subject 1 (Supplemental Fig. 1; supplemental materials are available at http://jnm.snmjournals.org).

**FIGURE 4.**
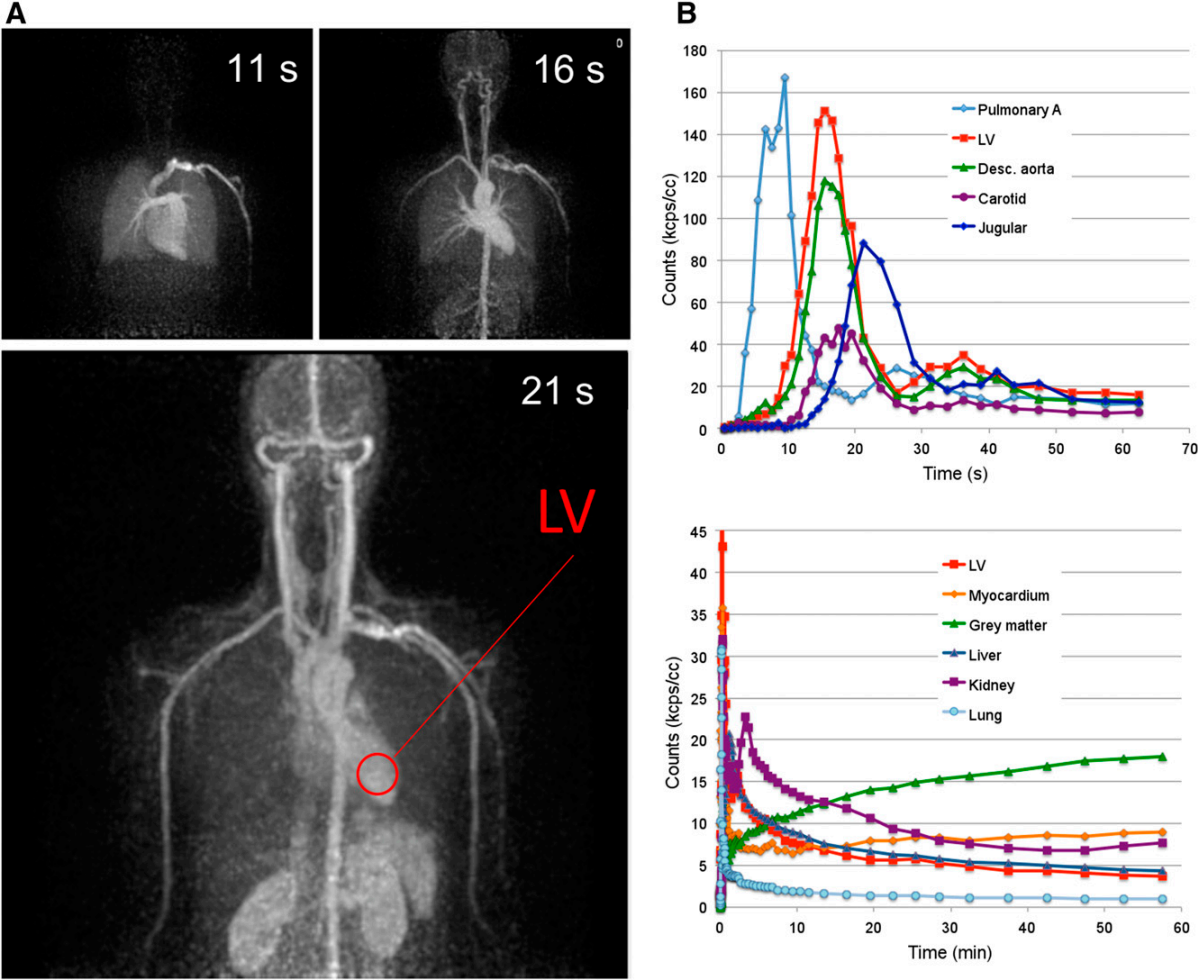
(A) ^18^F-FDG PET maximum-intensity projections of subject 7, each 1 s in duration, at 3 time points from dynamic scan. (B) Time–activity curves of blood input function measured at several vessels over first minute after injection, and time–activity curves of major organs over first hour after injection. LV = left ventricle.

Subject 7 was injected with a rapid bolus of ^18^F-FDG (∼2 s) inside the PennPET and scanned for 1 h to study the early kinetics of ^18^F-FDG with particular attention to the blood input function. Figure 4 shows representative time frames in the initial uptake, each 1 s in duration; a video of the dynamic scan is seen in Supplemental Video 1. The video includes 70 reconstructed-image frames ranging from 1 s to 5 min and shows the time–activity curves for blood input function and major organs. This fine temporal sampling, in combination with the excellent image quality of the PennPET, allows the identification of vascular structures as signal appears within the vessels. For example, the arterial vasculature of the head and neck is seen at 16 s, followed by the venous vasculature at 21 s. Figure 4B shows the blood input function measured in several vessels and the left ventricle. These time–activity curves demonstrate the expected path of ^18^F-FDG from the pulmonary artery to the left ventricle and into the systemic circulation, with low sampling noise. The effects of radiotracer dispersion and partial-volume averaging are apparent. Also shown in Figure 4B are the time–activity curves of major organs, illustrating the ability to measure all simultaneously.

### Clinical Subjects

**FIGURE 5.**
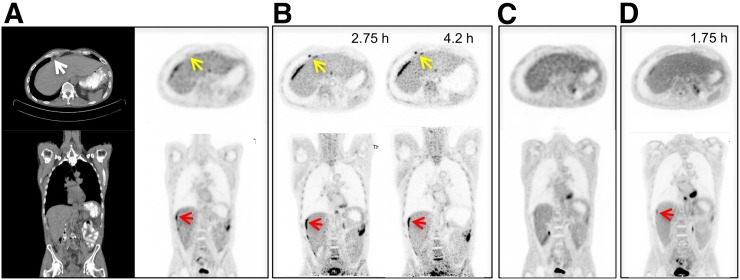
Clinical ^18^F-FDG PET/CT images (transverse and coronal) from subject 5, with metastatic colon cancer, acquired with standard clinical protocol. (B) PennPET image acquired 2.75 and 4.2 h after injection (10-min scans). Matched coronal and transverse slices are shown. Red arrows denote perihepatic disease; yellow arrows denote epiphrenic lymph node. (C) Follow-up clinical scan at 3 mo (subject 10). (D) Corresponding PennPET image (20-min scan) demonstrating improvement in perihepatic disease and epiphrenic lymph node.

Three clinical subjects were scanned on the PennPET after undergoing SOC PET to allow for direct comparison between the scanners. The default clinical reconstruction algorithm was used for the SOC PET studies. A patient with metastatic colon cancer was scanned twice on the PennPET with ^18^F-FDG, before (subject 5) and after treatment (subject 10) (Fig. 5). On both PennPET scans, perihepatic disease is more conspicuous than on the SOC PET scan, in part because of clearance of ^18^F-FDG from the nondiseased adjacent liver. The PennPET scan also clearly demonstrates an ^18^F-FDG–avid epiphrenic (near the diaphragm) lymph node on the baseline scan that was not identified on the SOC scan.

**FIGURE 6.**
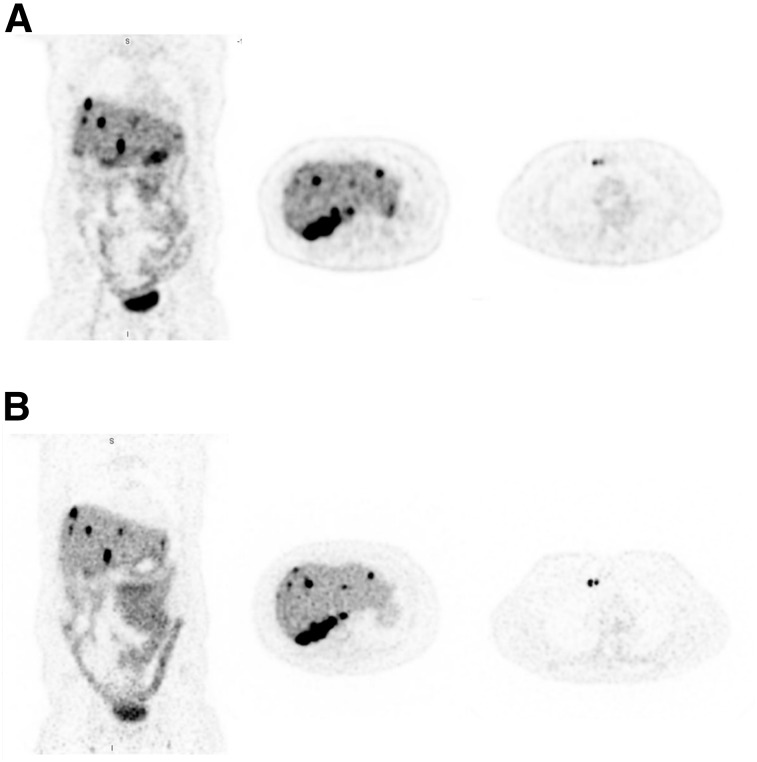
(A) SOC ^68^Ga-DOTATATE PET images (coronal and transverse) of subject 8, with metastatic neuroendocrine tumor. (B) Coronal and transverse images from same subject on PennPET acquired 3.5 h after injection (20-min scan).

Subject 8, with metastatic neuroendocrine cancer undergoing a ^68^Ga-DOTATATE PET study, was scanned to compare ^18^F-FDG with a radiotracer that has a shorter half-life and a lower administered activity (half-life, 68 min for ^68^Ga vs. 110 min for ^18^F). The activity at the time of scanning on the PennPET (3.5 h after injection) was one fifth that at the time of the clinical scan (65 min after injection), effectively corresponding to an injected activity of about 30 MBq. Nonetheless, qualitative inspection of the 2 scans, shown in Figure 6, demonstrates comparable diagnostic image quality between the PennPET scan and the clinical scan. Given the high cost and limited availability of ^68^Ga-DOTATATE, scanning at a much lower activity may have practical implications.

### Research Subjects

**FIGURE 7.**
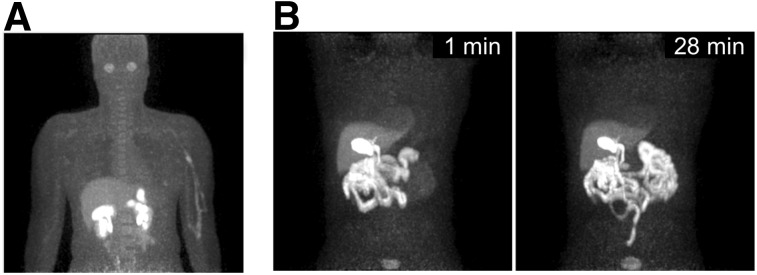
(A) Maximum-intensity projection (3-min scan) of ^18^F-labeled NOS PET (subject 6). (B) Maximum-intensity projections of ^18^F-fluortriopride PET (subject 9) for 1-min duration shown at 1 min (left) and 28 min (right) after drinking Ensure (Abbott Laboratories) to stimulate emptying of radiotracer from gallbladder.

The 2 research subjects were scanned on the PennPET following protocol-specific research PET scans with experimental research radiotracers. A representative image (3 min scan) of subject 6 is shown 2 h after intravenous administration of 226 MBq (6 mCi) of ^18^F-NOS (Fig. 7A). Whole-body imaging revealed unexpected ocular uptake in this study, which was excluded from the FOV of the standard research scan. Subject 9 was injected with ^18^F-fluortriopride and scanned dynamically for 30 min, with images centered over the gallbladder (Fig. 7B). Representative images (1-min scans) demonstrated mild gallbladder emptying over time, underscoring potential uses for the PennPET in dosimetry studies. These research studies demonstrate unique PennPET capabilities for PET research investigation, motivating further studies with these and other radiotracers.

## DISCUSSION

Initial human imaging studies on the prototype PennPET demonstrate the diversity of applications possible for a sensitive whole-body imager. These studies provide proof of concept for several of the projected applications of the PennPET (4,5). For clinical use, the PennPET can produce higher-quality images more quickly than current commercial scanners or comparable images with a significantly reduced activity. As a research tool, the expanded axial FOV of the PennPET not only allows for greater axial coverage but also enables dynamic whole-body imaging to benefit kinetic analysis studies. The increased sensitivity afforded by the long axial FOV allows delayed imaging, which may improve lesion detection and enable fundamental biologic insights.

Initial qualitative comparison shows ^18^F-FDG PennPET images to be of superior quality to SOC PET images when performed with similar scan durations as shown in Figure 1. These improvements in image quality translate to better delineation of sites of disease in subject 5, with metastatic colon cancer, on the PennPET scan than on the SOC scan, noting that the PennPET was performed later (Fig. 5). Perihepatic disease was more conspicuous on the PennPET images, and an epiphrenic lymph node was visualized only with the PennPET. More accurate delineation of disease may have treatment implications for both ^18^F-FDG and other tracers. Beyond oncology, imaging small brain structures may benefit from better count statistics due to the large acceptance angle of the PennPET, as shown in Figure 2. It is perhaps more noteworthy that the large axial coverage of the PennPET presents a unique opportunity to study brain–body interactions with dynamic imaging protocols.

Compared with commercially available PET scanners with a standard axial FOV, long-axial-FOV imagers such as the PennPET can produce images of comparable quality in much less time. The subsampled data from subject 1 showed that a 2-min scan on the PennPET was of comparable quality to that achieved in 16 min on the clinical scanner. This 8-fold decrease in scan time could increase patient throughput in a busy clinic and aid patient comfort. The images of the liver in Figure 1 demonstrate low noise and uniformity in subsampled images obtained in less than 1 min, suggesting that detectability of small lesions would be preserved at very short scan times. Such short scans could be leveraged to obtain breath-hold PET images, which may benefit thoracic imaging (16). Furthermore, for pediatric indications, scan times sufficiently short to forego sedation would improve safety and decrease the cost and complexity of imaging (17). For specific applications, scan time could be tailored to the clinical need for disease characterization.

Similarly, the increased sensitivity of the PennPET also facilitates scanning lower activities of radiotracer than are typically used, without compromising image quality. Comparable images were obtained with the PennPET with effectively one fifth the DOTATATE activity used for the clinical scan (Fig. 6). Images with lower activity may prove beneficial for pediatric patients (18), as well as for radiotracers of limited supply, including those for research and clinical care. With limited availability of ^68^Ga from a ^68^Ge/^68^Ga generator (19) and research efforts to produce ^68^Ga from a cyclotron, the PennPET may be used in specialized centers to maximize the clinical availability of this radiotracer. Finally, the increased sensitivity could be used to better image the rare positron from the decay of ^90^Y (20) or the low positron fraction of ^89^Zr with cell tracking.

The increased sensitivity of the PennPET enables imaging at later time points, exploiting washout of ^18^F-FDG from normal tissues and trapping in malignancy. This ability is seen clearly with the perihepatic disease in subject 5. The sensitivity with which lesions are detected may consequently improve (21). Similarly, delayed imaging of gliomas improves distinction between tumor and normal gray matter because of faster washout of ^18^F-FDG from the gray matter (7). Markedly delayed imaging of ^18^F-FDG beyond 10 half-lives with the PennPET was performed for 3 subjects (1, 3, and 7) and clearly demonstrated washout of ^18^F-FDG from the brain, providing the most definitive evidence of the existence of the dephosphorylation constant, *k*_4_, in a human image. Spence et al. previously estimated *k*_4_ in gliomas and in normal brain with imaging up to 8 h (7). Berg et al. demonstrated washout from the brain in rhesus monkey studies (22). We are currently pursuing kinetic analysis studies to estimate *k*_4_ over the extended period of imaging. For PET dosimetry applications, more accurate delayed scans can better estimate the behavior of the tail of the time–activity curve, with resultant improvements in dosimetry estimates. An example of a dosimetry application was shown for ^18^F-fluortriopride.

Dynamic whole-body scanning with the PennPET can benefit kinetic analysis by simultaneously capturing structures outside a standard axial FOV, including sites of disease, relevant normal organs, and an input function. As shown in the time–activity curves for subject 7 (Fig. 4), the fine temporal sampling of PennPET allows relatively noise-free input curves, even capturing recirculation of radiotracer after the initial bolus. Comparison of vessels as radiotracer travels from the heart reveals significant partial-volume and dispersion effects. Having the left ventricle always within the FOV provides a validated image-derived input function (23), possibly obviating sophisticated correction techniques (24) or direct arterial sampling. The inclusion of such an input function could be used to estimate first-pass uptake of ^18^F-FDG in order to estimate tumor perfusion (25) and further characterize disease.

There are some limitations to these early human studies on this novel scanner. These studies were performed in a prototype 3-ring configuration. A separate commercial CT scanner was used for attenuation correction, necessitating image registration. As mentioned, the PennPET will soon be expanded with additional detector rings for a larger axial FOV, and an integrated CT scanner will then be installed to improve efficiency and CT coregistration. Quantification of radiotracer uptake at very delayed time points has proved challenging, especially for structures with very low activity relative to background activity. The challenge of such quantification will require a careful investigation of the accuracy of our data correction methods, especially background correction. Lastly, physiologic changes in subjects over extended periods—such as from eating, insulin release, and exertion—were not controlled for in this study and may confound interpretations of late ^18^F-FDG kinetics.

## CONCLUSION

These first human studies of the large-axial-FOV PennPET validate the successful implementation of many of the key design components (related to data acquisition and reconstruction of large datasets) described in our companion paper (6). Both clinical and research examples were provided, underscoring the power and versatility of the sensitive scanner. Future investigations will examine the benefits of the full device with an even larger axial FOV and will refine quantitative methods for analysis, optimize imaging protocols, and study novel applications, including dual-tracer imaging.

## DISCLOSURE

This work was supported by NIH R01-CA206187, R33-CA225310, R01-CA113941, and KL2TR001879. Support for development of the PennPET was also received from Philips Healthcare and from the Department of Radiology at the University of Pennsylvania. The research studies included in this article were supported by NIH R01-DA029840 and by a TAPITMAT-TBIC grant from the University of Pennsylvania. No other potential conflict of interest relevant to this article was reported.

## KEY POINTS

**QUESTION:** How does larger axial coverage of a PET instrument lead to benefits for human imaging?
**PERTINENT FINDINGS:** The clinical studies demonstrated excellent image quality and potential for imaging with lower activity and shorter scan durations. The studies also demonstrated potential for very delayed imaging and measurement of multiorgan kinetics.
**IMPLICATIONS FOR PATIENT CARE:** The high sensitivity and large axial coverage of the PennPET whole-body imager will lead to benefits for clinical ^18^F-FDG studies and enable translational research that leverages the ability to measure kinetics in multiorgan systems.
