# Supplementary figures and images for: PennPET Explorer: Human Imaging on a Whole-Body Imager
Source: J Nucl Med. 2020 Jan;61(1):144–51. doi: 10.2967/jnumed.119.231845 (PMC6954463; doi:10.2967/jnumed.119.231845)

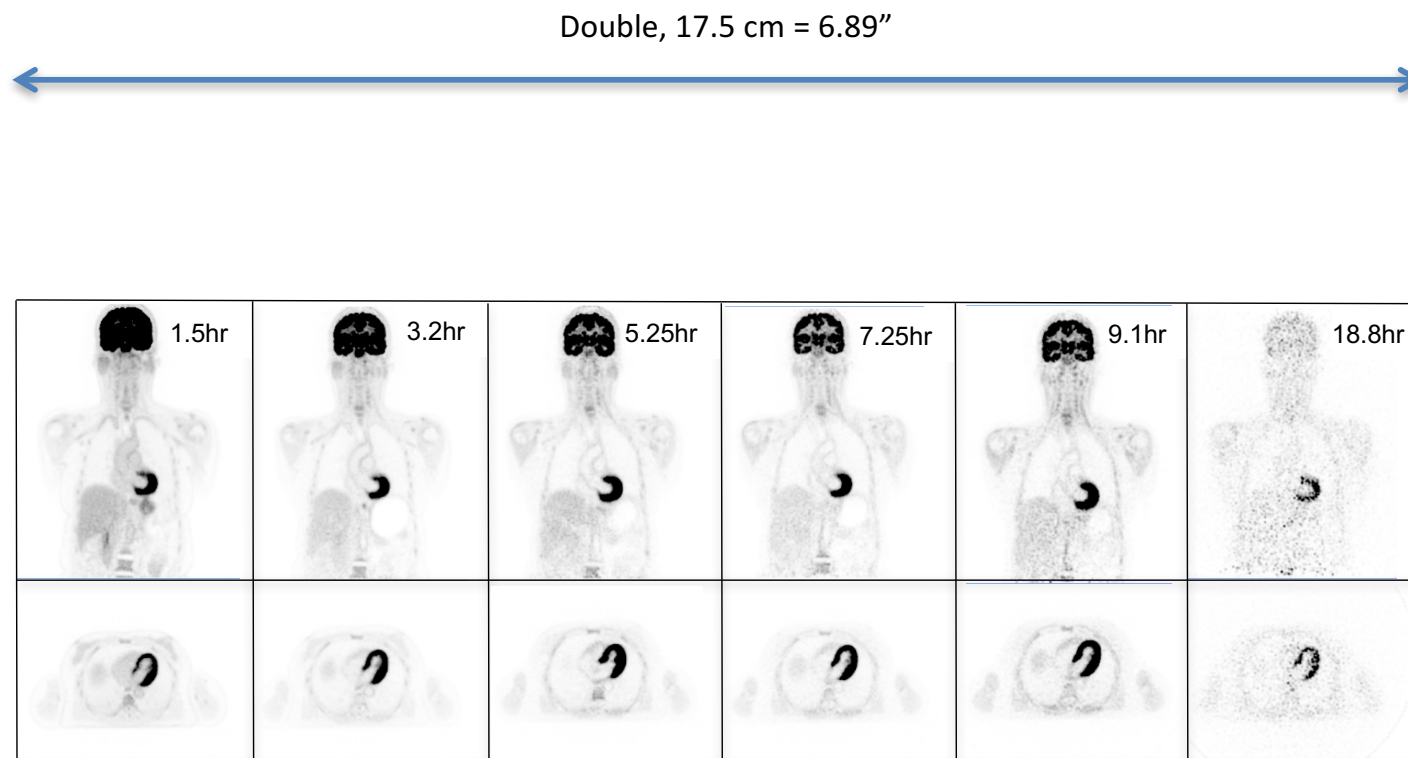

Supplemental Figure 1

Supplement: Supplementary file 1 [file jnm231845SupplementaryData.pdf]
